# Gilles de la tourette’s syndrome and psychosis: A family case study

**DOI:** 10.1192/j.eurpsy.2021.1709

**Published:** 2021-08-13

**Authors:** K. Konstantinova, K. Baidina, M. Losevich

**Affiliations:** Medicine, University of Latvia, Riga, Latvia

**Keywords:** family case, comorbidities, psychosis, Gilles de la Tourette’s syndrome

## Abstract

**Introduction:**

Tourette’s syndrome (ST) is a neuropsychiatric disorder that presents with combination of motor and vocal tics for at least one year time.Only few cases of comorbidity with psychotic disorder has been described.

**Objectives:**

We present a case report of a patient with ST, obsessive compulsive disorder, posttraumatic stress disorder that resulted in chronic schizophrenia-like psychosis, and family hystory of tics and psychosis.

**Methods:**

A case – based family study, literature review and statistic data analysis.

**Results:**

The patient (male, born in 1997 otherwise healthy)presented at the age of 6 with spitting.He subsequently progressed with severe motor tics, vocalizations, coprolalia, impulsivity, destructivity, repetitive motor rituals.No treatment showed to be efficacious and safe. He dropped out of the school, the family has to move to the rural area; his social withdrawal was intensified by psychotrauma (assaulted by police officer due to seemingly disorderly conduct). At the age of late adolescence he started to make fantastic statements. Later on he admitted having visual and audial hallucinations and responding to them; the Kandinsky–Clérambault syndrome was detected. Symptoms and exitement are partially controlled by diazepam and clozapine; the patient needs assistance in all routines of self – care.The patient’s mother has a mild form of motor tics; her mother developed persistent delusions in her late thirties (without disorders of perception and social disfunction).

**Conclusions:**

The study demonstrantes genetic interconnecting between TS, tics and psychosis; hyperactivity in the dopaminergic system of the brain may be involved in all three disorders. National statistics of TS have to be reviewed and improved.

**Disclosure:**

No significant relationships.

